# Diabetes-Associated Neuroplastic Changes in Neuropeptide Y-Immunoreactive Enteric Neurons Along the Porcine Gastrointestinal Tract

**DOI:** 10.3390/ijms27177534

**Published:** 2026-08-23

**Authors:** Michał Bulc, Barbara Jana, Katarzyna Palus

**Affiliations:** 1 Department of Clinical Physiology, Faculty of Veterinary Medicine, University of Warmia and Mazury in Olsztyn, Oczapowskiego St. 13, 10-718 Olsztyn, Poland; katarzyna.palus@uwm.edu.pl; 2 InLife Institute of Animal Reproduction and Food Research, Polish Academy of Science, Trylińskiego St 18., 10-683 Olsztyn, Poland; b.jana@pan.olsztyn.pl

**Keywords:** diabetes mellitus, enteric nervous system, neuropeptide Y, diabetic enteric neuropathy, neuroplasticity, pig

## Abstract

Diabetes mellitus is frequently associated with gastrointestinal dysfunction, in which alterations in the enteric nervous system (ENS) are considered important contributing factors. Neuropeptide Y (NPY) is a key enteric neuromodulator involved in the regulation of gastrointestinal motility, secretion, and blood flow; however, its response to diabetes remains insufficiently characterized, particularly in large animal models. This study investigated the effect of experimental diabetes on the distribution of NPY-immunoreactive enteric neurons in the porcine gastrointestinal tract. Diabetes was induced in juvenile female pigs by streptozotocin administration. Six weeks later, the stomach, duodenum, jejunum, ileum, and descending colon were collected, and the population of NPY-immunoreactive neurons in the myenteric and submucosal plexuses was evaluated by double-label immunofluorescence. Experimental diabetes significantly increased the population of NPY-immunoreactive neurons in the myenteric plexus of the stomach, jejunum, ileum, and descending colon, whereas no statistically significant changes were detected in the duodenum after correction for multiple comparisons. In the submucosal plexuses, significant increases were restricted to the inner and outer submucosal plexuses of the descending colon. These findings demonstrate that diabetes induces region- and plexus-specific neurochemical plasticity of NPY-immunoreactive enteric neurons in pigs. Given the predominantly inhibitory effects of NPY on gastrointestinal motility and secretion, together with its vasoconstrictive actions, the observed changes may contribute to altered neural regulation of gastrointestinal function during diabetes and may represent one of the mechanisms involved in diabetic gastroenteropathy.

## 1. Introduction

The digestive tract of mammals exhibits a considerable amount of anatomical diversity. Its structure is adapted to the specific dietary intake and type of diet in individual species [1,2]. The regulation of the gastrointestinal tract is also unique and characteristic of this system. In contrast to most internal organs, individual sections of the gastrointestinal tract have, in addition to the classic sympathetic and parasympathetic innervation, an intramural system of interconnected neurons and their processes forming the enteric nervous system (ENS) [3,4]. It is currently known that the organisation of the ENS is species-specific. In large animal species, such as pigs and humans, the ENS is distributed throughout the gastrointestinal tract and is organized into the myenteric plexus (MP) and submucosal plexuses (SP) [5]. The former is located between the longitudinal and the circular muscular layers, and the latter is near the mucosal basement membrane. Each plexus is formed from a species-specific number of ganglia within which nerve cells are located. In the large mammalian species mentioned above, the submucosal plexus is divided into two parts, one of which, called the inner submucosal plexus (ISP), is located in the immediate vicinity of the outer side of the muscularis mucosae, and the other is known as the outer submucosal plexus (OSP), and is found on the inner side of the circular muscular layer [6,7,8].

The nerve cells, regardless of their location (central, peripheral, or enteric), communicate with each other through biologically active substances known as neurotransmitters. Not only are these substances used to transmit nerve impulses, but they also modify neuronal function, in which case they are referred to as neuromodulators [9,10]. One of the most widely distributed and biologically active neuropeptides acting as a neuromodulator is neuropeptide Y (NPY). NPY was first isolated from porcine brain in 1982 [11]. It is one of the most abundant neuropeptides in both the peripheral and central nervous systems and exhibits an extremely conserved primary structure among all mammals studied to date [12]. Its presence in enteric neurons has been confirmed in humans and other mammalian species [13,14]. NPY has been identified in the perikarya of neurons forming both the myenteric and submucosal plexuses, as well as in the enteric nerve fibres. It should be emphasised that the population of NPY-expressing neurons in the porcine gastrointestinal tract is relatively small and does not exceed 5%, according to available studies [15]. Functionally, NPY exerts predominantly inhibitory effects on gastrointestinal motility and secretory activity. In addition, NPY strongly influences the tone of gastrointestinal blood vessels, increasing vascular resistance and thereby reducing local blood flow [16,17]. NPY was selected for this study because of its involvement in neuroprotection, modulation of gastrointestinal motility, and autonomic regulation. NPY expression may be particularly responsive to metabolic and oxidative stress, including conditions associated with hyperglycaemia, suggesting its potential involvement in adaptive or pathological neurochemical remodelling of the ENS during diabetes. Due to the complex nervous regulation of the gastrointestinal tract—which involves motor activity, blood flow, secretory, and resorptive processes—the function of this system is often impaired in the course of many diseases [18]. One of them is diabetes, in the course of which many gastrointestinal disorders occur, especially in patients with elevated glycated haemoglobin levels. These are referred to as diabetic gastroenteropathy. Its cause is usually multifactorial, and it may be induced by, inter alia, autonomic neuropathy. Approximately 75% of patients with diabetes experience various forms of gastrointestinal dysfunction, which are probably a consequence of impaired intestinal motility [18,19,20,21]. Typical gastrointestinal symptoms include dysphagia, reflux, early satiety, nausea, abdominal pain, diarrhoea or constipation. The pathogenesis of gastrointestinal dysfunction in the course of diabetes remains a subject of research, and the role of the enteric nervous system and neurotransmitters has gained importance in recent years. In various neurodegenerative, inflammatory, infectious, and toxicological disorders of the gastrointestinal tract, functional adaptation occurs in ENS neurons [22,23]. This process is reflected in quantitative changes in the number of neurons immunoreactive for different biologically active substances. The pattern of these changes depends on both the region of the gastrointestinal tract and the specific substance examined. A previous study in pigs with chemically induced diabetes demonstrated pronounced neuroplasticity in the intramural neurons of the gastrointestinal tract [24,25,26]. Despite accumulating evidence, important questions remain regarding the functional significance of diabetes-induced neurochemical remodelling within the ENS. Specifically, it remains unclear whether alterations in NPY expression represent primarily an adaptive and potentially neuroprotective response to metabolic stress or form part of the pathological neurochemical remodelling associated with diabetic gastrointestinal dysfunction. Therefore, the aim of this study was to determine quantitative changes in NPY-immunoreactive neurons in the enteric nervous system of the stomach and intestines of pigs with streptozotocin-induced diabetes accompanied by hyperglycaemia.

Of the many enteric neurotransmitters, NPY was chosen as the main target because of its involvement in metabolic regulation and neuroprotective mechanisms, as well as its responsiveness to metabolic and oxidative stress. Furthermore, NPY participates in the regulation of neuroimmune communication and intestinal microvascular tone, making it a particularly relevant neuropeptide for investigating enteric neurochemical plasticity associated with diabetes. This study contributes to our understanding of the role of peptidergic neuromodulators, such as NPY, in gastrointestinal dysfunction associated with diabetes. This is particularly important given the lack of detailed quantitative data on NPY-immunoreactive enteric neurons in diabetic gastroenteropathy. To date, such analyses have been performed mainly in rodent models of diabetes.

Although rats are widely used in biomedical research because of their low breeding costs and the ease of genetic manipulation, the domestic pig represents a more relevant translational model for studies of the gastrointestinal tract. The porcine digestive system closely resembles that of humans with respect to anatomical structure, physiological function, and pathological responses. In particular, pigs and humans share a comparable relative intestinal length, a well-developed colon, a similarly structured single-chambered stomach, and a comparable organization of digestive glands. Moreover, both species are omnivorous and process nutrients in a similar manner. The digestion of fats, proteins, and carbohydrates in pigs therefore more closely reflects human physiology than that of rodents [27,28]. In addition to anatomical and dimensional similarities, porcine models have significant physiological advantages over rodents in the study of metabolic and gastrointestinal diseases. Pigs share closer similarities with humans regarding omnivorous diets, metabolic regulation, gastrointestinal transit times and the cytoarchitecture of the enteric nervous system. This makes porcine models particularly suitable for translating findings related to neurochemical plasticity into clinical gastroenterology.

## 2. Results

### 2.1. Validation of the Experimental Diabetes Model

After acclimatisation and before the start of the actual experiment, the glucose concentration in pigs in both groups was 5.01 ± 0.10 mmol/L in the control group and 5.03 ± 0.10 mmol/L in the experimental group, respectively. Throughout the entire experiment, the average glucose concentration in the control group animals, determined before morning feeding, remained within the physiological norm and amounted to 5.09 ± 0.14 mmol/L. In contrast, in the animals in the experimental group, a steady increase in the blood glucose concentration was observed, as a consequence of damage to the pancreatic beta cells. Approximately one week into the experiment, the average glucose concentration in animals in this group was four times higher than in animals in the control group, and amounted to 20.58 ± 0.55 mmol/L. The exact glucose concentration levels are provided in Table 1. Throughout the entire experiment, the animals were under constant veterinary supervision. Despite the significantly elevated glucose concentration, pigs in the experimental group remained in good general condition throughout the entire experiment. In addition, no glucose concentration correction was performed on animals in this group by insulin injections.

### 2.2. Distribution of NPY-Immunoreactive Neurons in the Corpus of the Stomach

**Figure 1:**
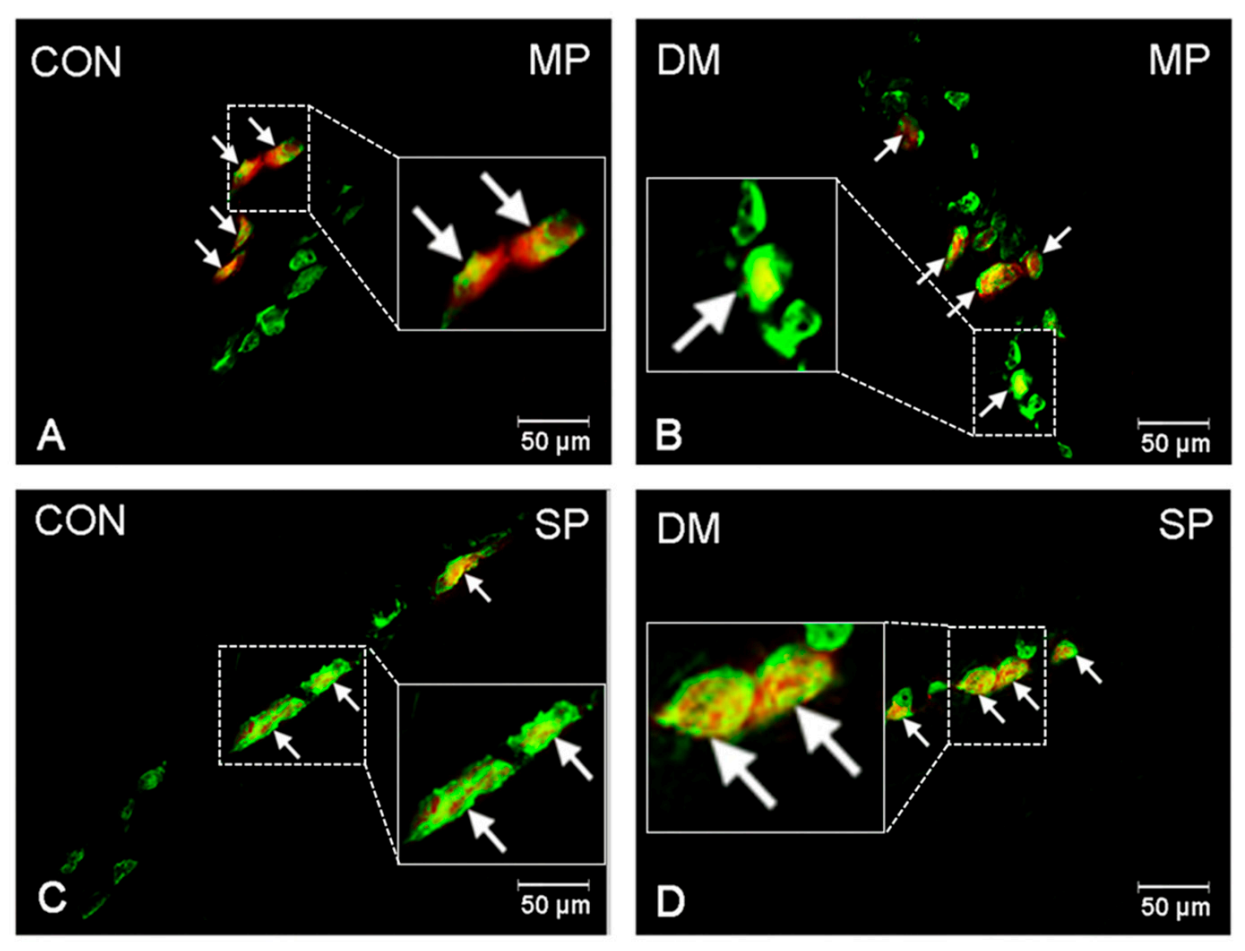
Myenteric plexus of the stomach in control (**A**) and diabetic (**B**) pigs. Submucosal plexus of the stomach in control (**C**) and diabetic (**D**) pigs. All images were obtained by digital superimposition of two fluorescence channels (green: Hu C/D, used as a pan-neuronal marker; red: NPY). Neurons showing co-localization of Hu C/D and NPY are indicated by arrows. Insets show higher-magnification views of the corresponding areas indicated by dashed boxes in the main images.

**Figure 2:**
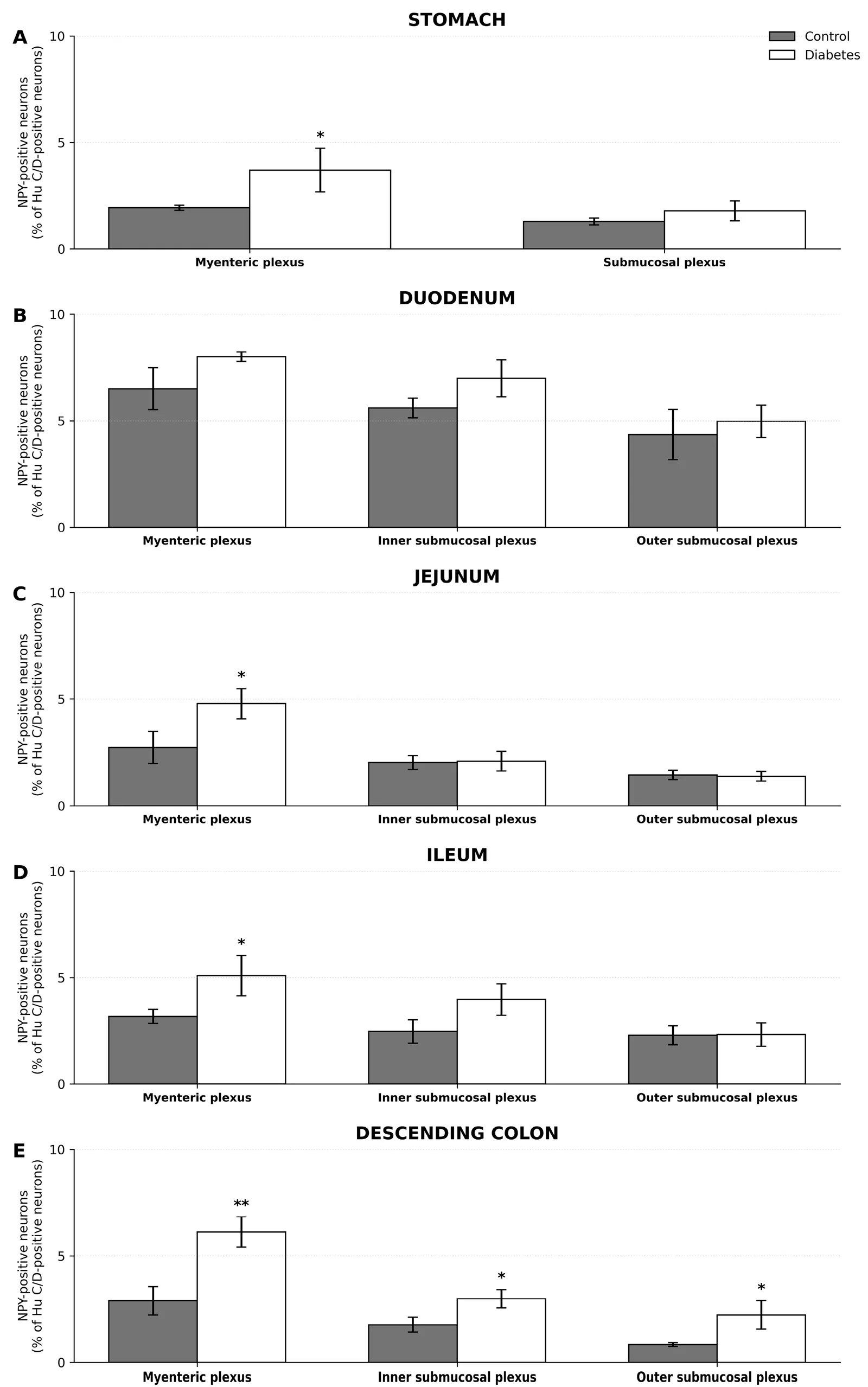
Percentage of NPY-immunoreactive neurons in the control (grey bars) and diabetic (white bars) groups in different regions of the gastrointestinal tract: (**A**) stomach; (**B**) duodenum; (**C**) jejunum; (**D**) ileum; and (**E**) descending colon. NPY-immunoreactive neurons are expressed as a percentage of the total number of Hu C/D-immunoreactive neurons. Data are presented as mean ± SD (*n* = 5 animals per group), with the individual animal considered the experimental unit. Statistical comparisons between the control and diabetic groups were performed using the independent Student’s *t*-test, with *p*-values adjusted for multiple comparisons using the Holm method. * *p* < 0.05 and ** *p* < 0.01 indicate statistically significant differences based on Holm-adjusted *p*-values.

In the corpus of the stomach, NPY-immunoreactive neurons constituted a relatively small proportion of the total Hu C/D-immunoreactive neuronal population in both the control and diabetic groups (*n* = 5 animals per group). In the myenteric plexus, the number of NPY-immunoreactive neurons was 1.93 ± 0.12% in the control group compared with 3.70 ± 1.02% in the diabetic group, corresponding to a 1.92-fold increase and a large effect size (Cohen’s *d* = 2.44). This difference remained statistically significant after correction for multiple comparisons (Holm-adjusted *p* = 0.044; Figure 1A,B and Figure 2A). In the submucosal plexus, NPY-immunoreactive neurons accounted for 1.29 ± 0.16% in the control group and 1.79 ± 0.47% in the diabetic group, corresponding to a 1.39-fold increase (Cohen’s *d* = 1.43). However, this difference was not statistically significant after Holm’s correction (adjusted *p* = 0.272; Figure 1C,D and Figure 2A).

### 2.3. Distribution of NPY-Immunoreactive Neurons in the Small Intestine

#### 2.3.1. Distribution of NPY-Immunoreactive Neurons in the Duodenum

**Figure 3:**
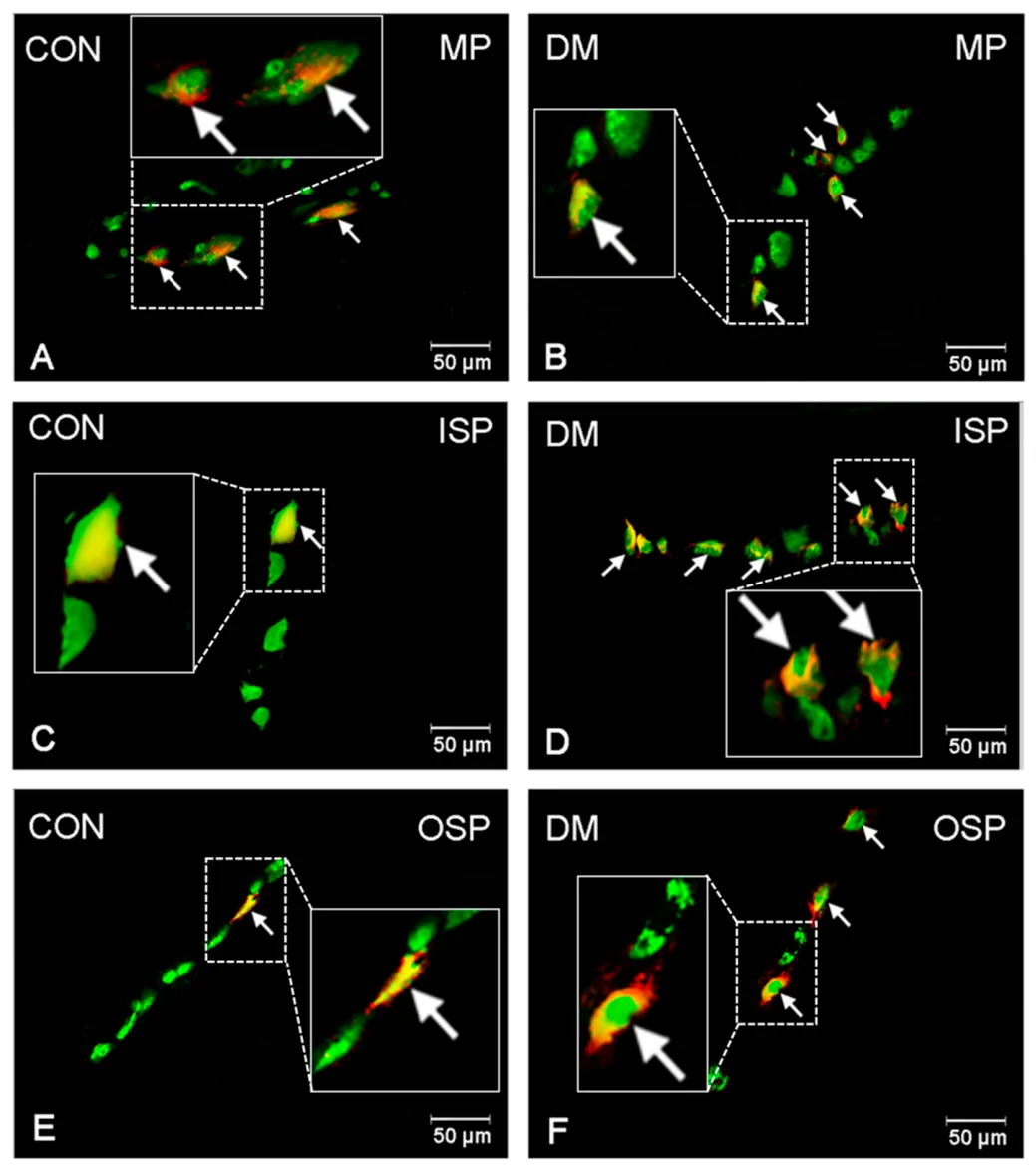
Myenteric plexus of the duodenum in control (**A**) and diabetic (**B**) pigs. Inner submucosal plexus of the duodenum in control (**C**) and diabetic (**D**) pigs. Outer submucosal plexus of the duodenum in control (**E**) and diabetic (**F**) pigs. All images were obtained by digital superimposition of two fluorescence channels (green: Hu C/D, used as a pan-neuronal marker; red: NPY). Neurons showing co-localization of Hu C/D and NPY are indicated by arrows. Insets show higher-magnification views of the corresponding areas indicated by dashed boxes in the main images.

In the duodenum, the highest population of NPY-immunoreactive neurons in control animals was observed in the myenteric plexus. NPY-immunoreactive neurons accounted for 6.50 ± 0.98% in the control group compared with 8.01 ± 0.22% in the diabetic group, corresponding to a 1.23-fold increase and a large effect size (Cohen’s *d* = 2.13). However, this difference did not remain statistically significant after correction for multiple comparisons (Holm-adjusted *p* = 0.069; Figure 2B and Figure 3A,B). In the inner submucosal plexus, the number of NPY-immunoreactive neurons was 5.60 ± 0.46% in the control group and 6.99 ± 0.87% in the diabetic group, corresponding to a 1.25-fold increase and a large effect size (Cohen’s *d* = 2.00). This difference was not statistically significant after Holm’s correction (adjusted *p* = 0.081; Figure 2B and Figure 3C,D). In the outer submucosal plexus, the corresponding values were 4.35 ± 1.18% and 4.97 ± 0.76%, respectively, representing a 1.14-fold increase (Cohen’s *d* = 0.62), with no statistically significant difference between the groups (Holm-adjusted *p* = 1.000; Figure 2B and Figure 3E,F).

#### 2.3.2. Distribution of NPY-Immunoreactive Neurons in the Jejunum

**Figure 4:**
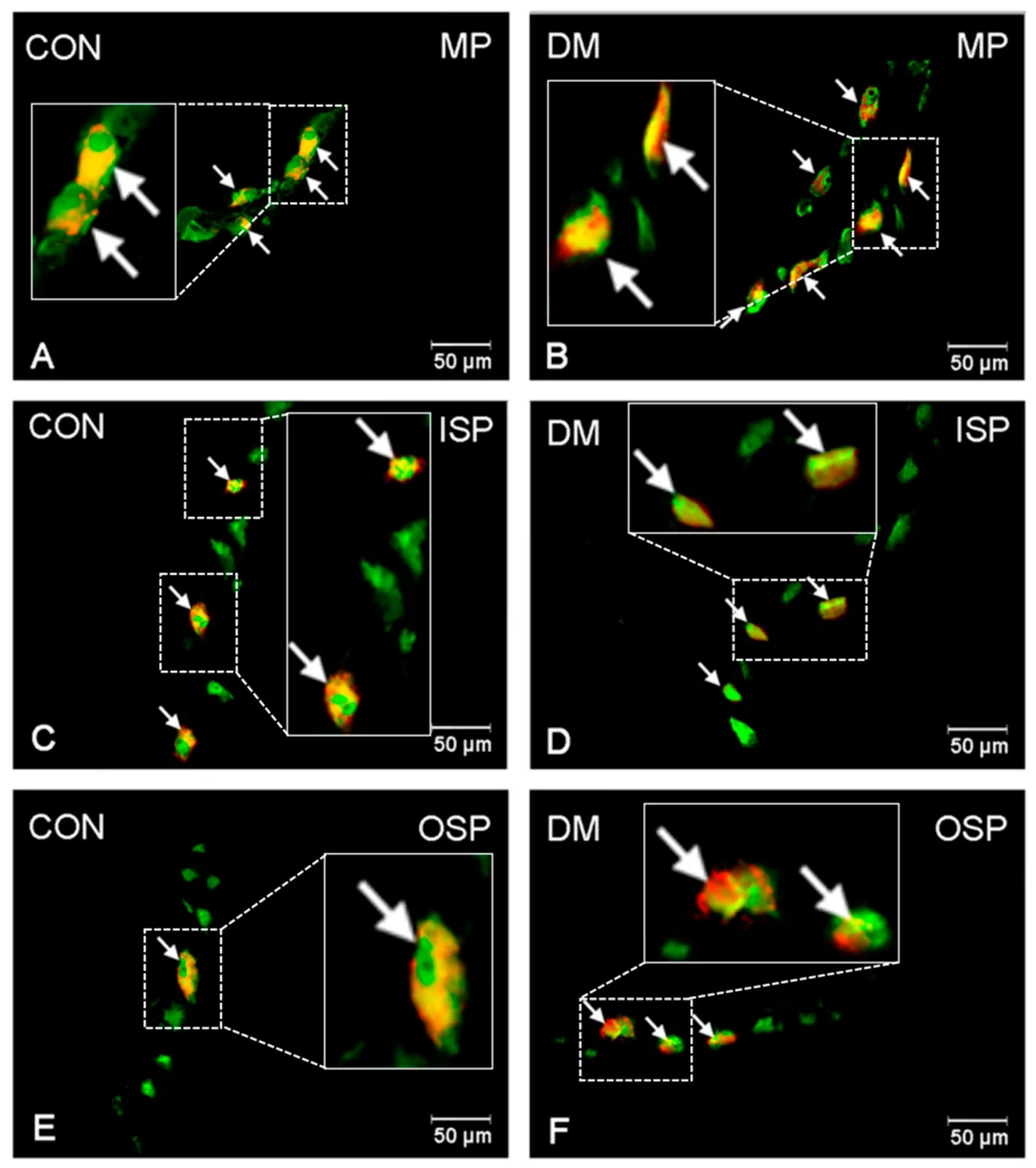
Myenteric plexus of the jejunum in control (**A**) and diabetic (**B**) pigs. Inner submucosal plexus of the jejunum in control (**C**) and diabetic (**D**) pigs. Outer submucosal plexus of the jejunum in control (**E**) and diabetic (**F**) pigs. All images were obtained by digital superimposition of two fluorescence channels (green: Hu C/D, used as a pan-neuronal marker; red: NPY). Neurons showing co-localization of Hu C/D and NPY are indicated by arrows. Insets show higher-magnification views of the corresponding areas indicated by dashed boxes in the main images.

In the jejunum, NPY-immunoreactive neurons constituted a relatively small proportion of the Hu C/D-immunoreactive neuronal population. In the myenteric plexus, NPY-immunoreactive neurons accounted for 2.73 ± 0.75% in the control group compared with 4.78 ± 0.71% in the diabetic group, corresponding to a 1.75-fold increase and a large effect size (Cohen’s *d* = 2.81). This difference remained statistically significant after correction for multiple comparisons (Holm-adjusted *p* = 0.024; Figure 2C and Figure 4A,B). In the inner submucosal plexus, the proportion of NPY-immunoreactive neurons was 2.03 ± 0.32% in the control group and 2.09 ± 0.45% in the diabetic group, corresponding to a 1.03-fold change (Cohen’s *d* = 0.15), with no statistically significant difference between the groups (Holm-adjusted *p* = 1.000; Figure 2C and Figure 4C,D). Similarly, in the outer submucosal plexus, the corresponding values were 1.45 ± 0.22% and 1.39 ± 0.23%, respectively, representing a 0.96-fold change (Cohen’s *d* = −0.27), with no statistically significant difference between the groups (Holm-adjusted *p* = 1.000; Figure 2C and Figure 4E,F).

#### 2.3.3. Distribution of NPY-Immunoreactive Neurons in the Ileum

**Figure 5:**
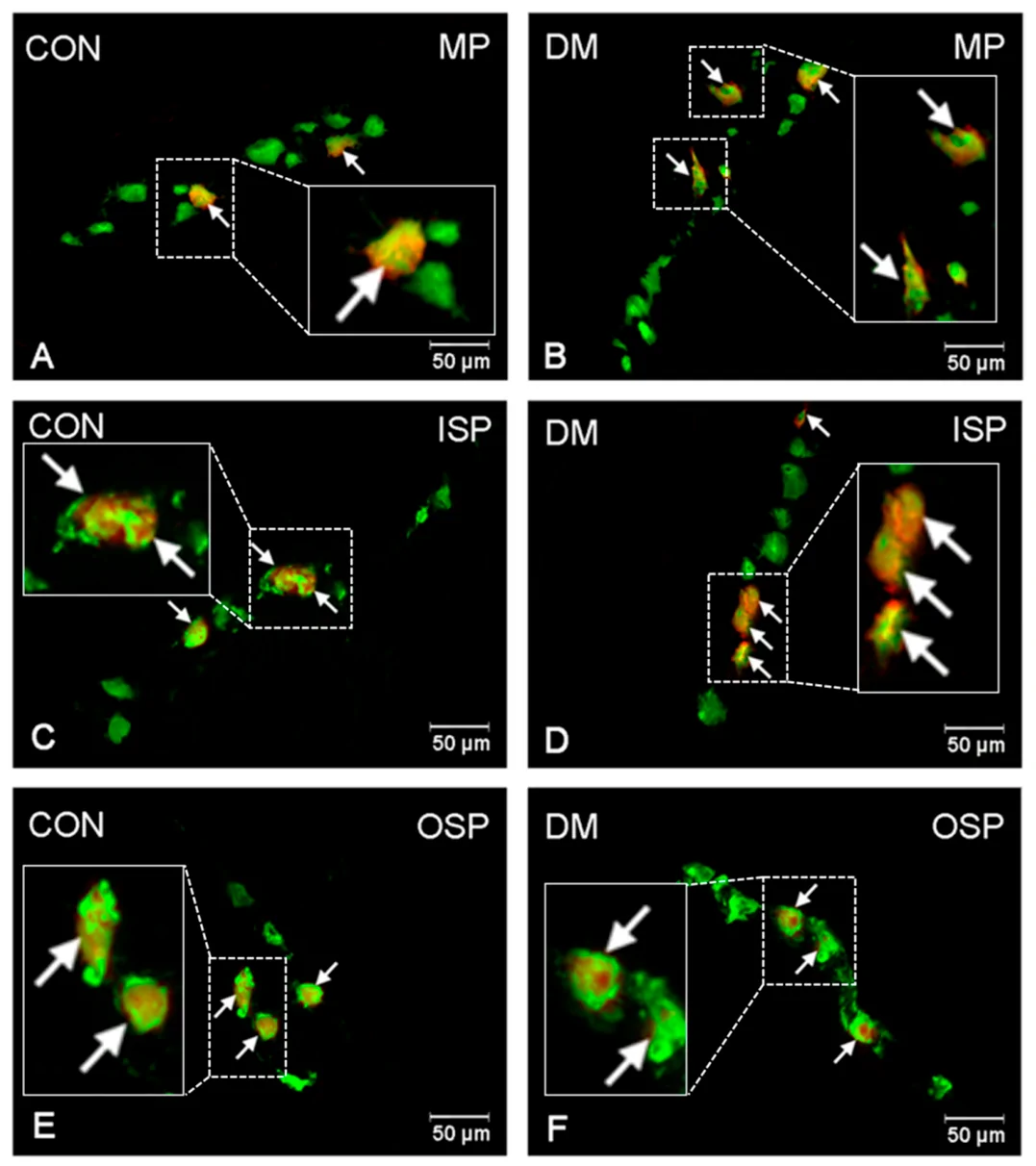
Myenteric plexus of the ileum in control (**A**) and diabetic (**B**) pigs. Inner submucosal plexus of the ileum in control (**C**) and diabetic (**D**) pigs. Outer submucosal plexus of the ileum in control (**E**) and diabetic (**F**) pigs. All images were obtained by digital superimposition of two fluorescence channels (green: Hu C/D, used as a pan-neuronal marker; red: NPY). Neurons showing co-localization of Hu C/D and NPY are indicated by arrows. Insets show higher-magnification views of the corresponding areas indicated by dashed boxes in the main images.

In the ileum, NPY-immunoreactive neurons were present in all examined intramural plexuses. In the myenteric plexus, NPY-immunoreactive neurons accounted for 3.18 ± 0.33% in the control group compared with 5.09 ± 0.94% in the diabetic group, corresponding to a 1.60-fold increase and a large effect size (Cohen’s *d* = 2.71). This difference remained statistically significant after correction for multiple comparisons (Holm-adjusted *p* = 0.027; Figure 2D and Figure 5A,B). In the inner submucosal plexus, the number of NPY-immunoreactive neurons was 2.47 ± 0.55% in the control group and 3.97 ± 0.74% in the diabetic group, corresponding to a 1.61-fold increase and a large effect size (Cohen’s *d* = 2.30). However, this difference did not remain statistically significant after Holm’s correction (adjusted *p* = 0.053; Figure 2D and Figure 5C,D). In the outer submucosal plexus, the corresponding values were 2.29 ± 0.44% and 2.33 ± 0.55%, respectively, representing a 1.02-fold change (Cohen’s *d* = 0.08), with no statistically significant difference between the groups (Holm-adjusted *p* = 1.000; Figure 2D and Figure 5E,F).

#### 2.3.4. Distribution of NPY-Immunoreactive Neurons in the Descending Colon

**Figure 6:**
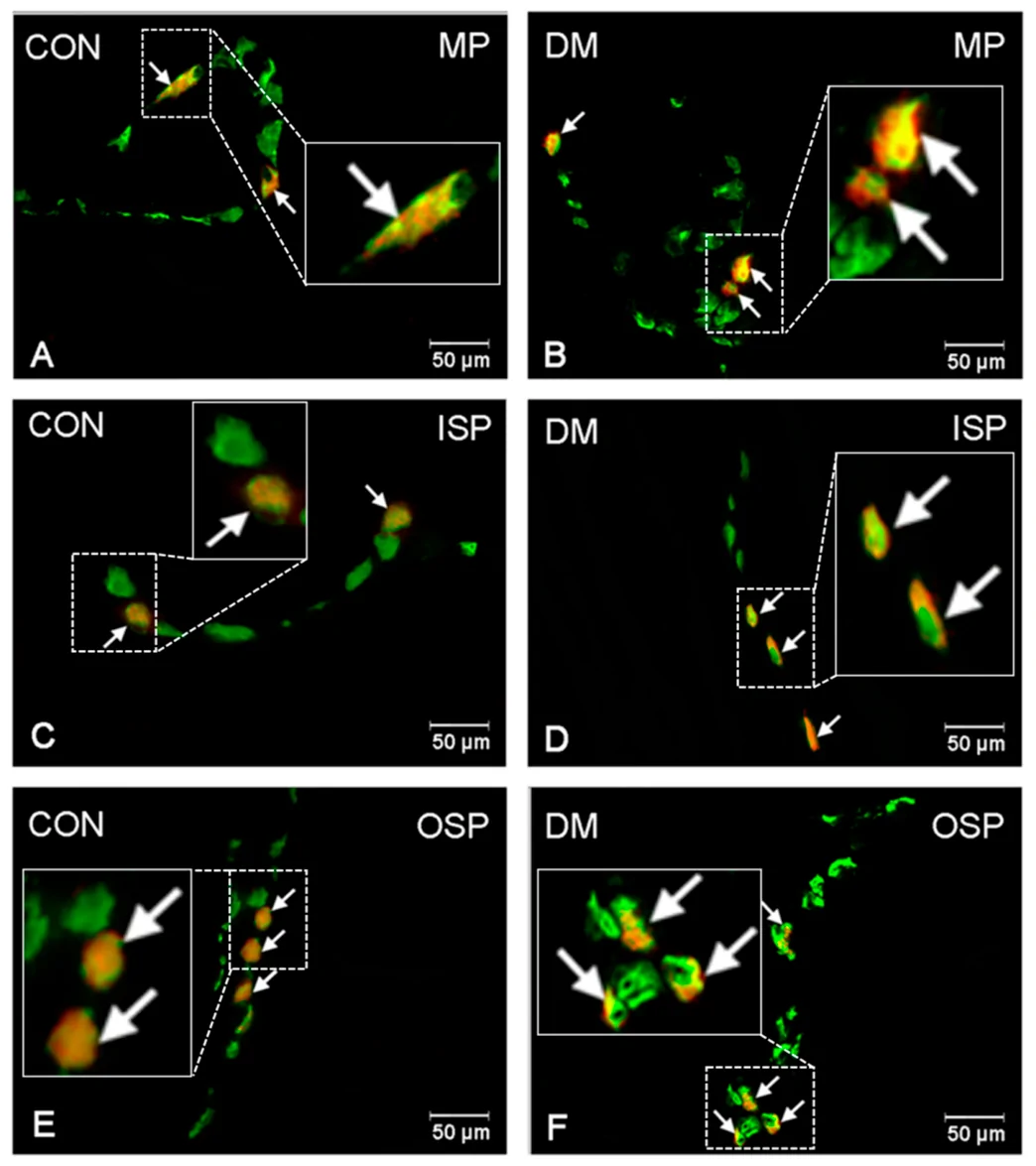
Myenteric plexus of the descending colon in control (**A**) and diabetic (**B**) pigs. Inner submucosal plexus of the descending colon in control (**C**) and diabetic (**D**) pigs. Outer submucosal plexus of the descending colon in control (**E**) and diabetic (**F**) pigs. All images were obtained by digital superimposition of two fluorescence channels (green: Hu C/D, used as a pan-neuronal marker; red: NPY). Neurons showing co-localization of Hu C/D and NPY are indicated by arrows. Insets show higher-magnification views of the corresponding areas indicated by dashed boxes in the main images.

The descending colon was the only region of the large intestine examined in the present study. In the myenteric plexus, NPY-immunoreactive neurons accounted for 2.89 ± 0.66% in the control group compared with 6.12 ± 0.71% in the diabetic group, corresponding to a 2.12-fold increase and a large effect size (Cohen’s *d* = 4.71). This difference remained statistically significant after correction for multiple comparisons (Holm-adjusted *p* = 0.001; Figure 2E and Figure 6A,B). Significant increases were also observed in both submucosal plexuses. In the inner submucosal plexus, the number of NPY-immunoreactive neurons increased from 1.77 ± 0.35% in the control group to 2.99 ± 0.43% in the diabetic group, corresponding to a 1.69-fold increase and a large effect size (Cohen’s *d* = 3.11; Holm-adjusted *p* = 0.015; Figure 2E and Figure 6C,D). In the outer submucosal plexus, the corresponding values were 0.84 ± 0.09% and 2.23 ± 0.67%, respectively, representing a 2.65-fold increase and a large effect size (Cohen’s *d* = 2.91). This difference was also statistically significant after Holm’s correction (adjusted *p* = 0.021; Figure 2E and Figure 6E,F).

## 3. Discussion

Hyperglycaemia is the main symptom of diabetes and occurs in both type 1 and type 2 diabetes. It results from partial or complete insulin deficiency associated with impaired function or progressive damage of pancreatic β cells [29]. In the present study, streptozotocin, a compound that inhibits DNA replication and exhibits selective cytotoxicity towards pancreatic β cells, was used to induce experimental diabetes. Its action results in a progressive decrease in insulin secretion and the development of sustained hyperglycaemia [30,31]. Therefore, streptozotocin is frequently used to experimentally induce diabetes, particularly in rodent models. In the present study, streptozotocin was successfully used to induce persistent hyperglycaemia in pigs. It should be emphasised that a single dose of 150 mg/kg b.w. was sufficient to produce this effect. This dose also falls within the range of streptozotocin doses used in experimental rodent models [32]. Prolonged hyperglycaemia poses a hazard to many tissues and organs, including neurons, particularly those of the peripheral nervous system, such as gastrointestinal intramural neurons [29]. The gastrointestinal tract is particularly susceptible to diabetes-related neuronal disturbances because of its complex innervation involving sympathetic and parasympathetic branches of the autonomic nervous system, as well as the enteric nervous system [3,4]. The pathomechanism of diabetic gastroenteropathy is complex. Importantly, before fully developed gastroenteropathy manifests with clinical symptoms such as diarrhoea, constipation, or disturbances in gastrointestinal transit, alterations may already occur at the cellular level [23]. In enteric neurons, one of the mechanisms of adaptation to pathological stimuli, including diabetes, involves changes in their neurochemical phenotype, which may be reflected by an increase or decrease in the population of neurons expressing particular biologically active substances [26].

The present study quantitatively assessed the proportion of intramural neurons immunoreactive for NPY in the porcine gastrointestinal tract under conditions of streptozotocin-induced diabetes associated with sustained hyperglycaemia. NPY is present in numerous neuronal systems of the brain, from the medulla oblongata to the cerebral cortex, as well as in the enteric neurons, including the secretory and inhibitory neuronal populations [33]. NPY is a substance commonly found throughout the gastrointestinal tract [34]. However, its distribution and the proportion of NPY-immunoreactive neurons depend on the gastrointestinal region, type of enteric plexus and, importantly, the animal species examined [34,35]. In the present study, NPY-immunoreactive neurons were detected in the intramural plexuses of all examined gastrointestinal regions. In the control animals, they constituted a relatively small population, with their proportion not exceeding 7% of Hu C/D-immunoreactive neurons. The highest proportion of NPY-immunoreactive neurons was observed in the duodenal intramural plexuses. In addition, the myenteric plexus in each examined gastrointestinal region contained a higher proportion of NPY-immunoreactive neurons than the corresponding submucosal plexuses. In the available literature, relatively few studies have described the exact percentage of NPY-immunoreactive e neurons within the porcine enteric nervous system [36]. The co-occurrence of NPY with serotonin in the submucosal plexuses of the small intestine has been described [37]. However, in the myenteric and submucosal plexuses of the corpus and pyloric region of the wild boar stomach, no co-occurrence of NPY with CART-immunoreactive neurons has been observed [38]. NPY-immunoreactive neurons represented a relatively large population in the rat small intestine, where their proportion in the myenteric plexus was estimated at 26%. An even more numerous population of NPY-immunoreactive neurons was noted in the submucosal plexuses of the ileum, where they accounted for approximately 36% [13]. A markedly smaller population of NPY-immunoreactive neurons was found in the submucosal plexuses of the mouse colon, where they accounted for approximately 8% [37]. Similarly, a high proportion of neurons expressing NPY, particularly in the small intestine, has been described in the guinea pig [35]. In contrast, other studies in rats reported considerably lower proportions of NPY-immunoreactive neurons. In the myenteric plexus of the small intestine, the proportion of these neurons was similar to that observed in the present study and averaged approximately 4% [13]. Taken together, previous studies indicate that the proportion of NPY-immunoreactive neurons in the gastrointestinal intramural plexuses is strongly species-dependent and additionally varies according to gastrointestinal region and plexus type. Nevertheless, relatively abundant populations of NPY-immunoreactive neurons are frequently found in the myenteric plexuses of the small intestine, which is consistent with the present findings, in which the highest proportions in control animals were observed in this part of the gastrointestinal tract.

A characteristic feature of the nervous system, including enteric neurons, is neuronal plasticity—a process that enables adaptation to physiological and pathological stimuli [38] through rearrangement of the biochemical machinery of neurons aimed at maintaining functional integrity. In the present study, enteric neurons were exposed to sustained hyperglycaemia [39], which was associated with a marked, but region- and plexus-specific, alteration in the proportion of NPY-immunoreactive neurons. After correction for multiple comparisons, significant increases were observed in the myenteric plexus of the stomach, jejunum, ileum, and descending colon, whereas no statistically significant changes were detected in the duodenum. Within the submucosal plexuses, significant increases were restricted to the inner and outer submucosal plexuses of the descending colon. These findings indicate that diabetes-associated NPY neurochemical plasticity is not uniform throughout the gastrointestinal tract but depends on both the anatomical region and the type of enteric plexus examined.

Quantitative alterations in NPY expression during diabetic enteropathy have previously been investigated primarily in rodent models [37,40,41], whereas, to the best of our knowledge, the pig was used for this purpose for the first time in the present study. In diabetic rats, NPY levels and the proportion of NPY-immunoreactive neurons have been reported to increase in the ileal myenteric plexus but remain unchanged in the colon and small intestinal submucosal plexuses [37,40,41], whereas diabetic mice exhibit increased NPY-ergic innervation in the colon, but not in the stomach or duodenum [37]. When comparing the present findings with these rodent models, notable species-specific differences emerge. Although diabetes-associated NPY-related neurochemical plasticity has been reported across species, rodent models display divergent, segment-dependent responses compared with the region- and plexus-specific pattern observed in the porcine gastrointestinal tract [42,43]. Particularly noteworthy in the present study was the involvement of the myenteric plexus in several gastrointestinal regions and the widespread response of both myenteric and submucosal plexuses in the descending colon. Given that the wall architecture and neurochemical organization of the porcine enteric nervous system more closely resemble those of humans than do those of small rodents, these differences further emphasise the potential translational value of large-animal models in studies of diabetic enteropathy.

The increased proportion of NPY-immunoreactive neurons observed in selected gastrointestinal regions in the present study suggests enhanced NPY expression within specific enteric neuronal populations. Although NPY release was not directly assessed, such neurochemical remodelling may alter NPY-mediated signalling within the gastrointestinal wall. NPY exerts its biological effects through binding to several subtypes of transmembrane Y receptors coupled to G proteins. Five Y receptors have been described in mammals, including humans: Y1, Y2, Y4, Y5, and Y6. Y1, Y2, and Y4 receptors have been identified in the colon and small intestine, and their expression has also been confirmed in epithelial cells and neurons of both the submucosal and myenteric plexuses [44,45]. After binding to its receptors within the gastrointestinal tract, NPY exerts numerous physiological effects. It participates in the regulation of gastric emptying and gastrointestinal transit, including the passage of intestinal contents from the ileum to the colon. NPY also inhibits gastric and pancreatic secretion and stimulates the absorption of water and electrolytes. Another important function of NPY, demonstrated in numerous studies, is modulation of immune cell activity, including macrophages, lymphocytes, and monocytes [35,44,46,47]. These cells express, among others, Y1 and Y2 receptors, and NPY signalling through these receptors may influence their activity and the release of inflammatory mediators. NPY may therefore play an important role in immune processes within the gastrointestinal tract, particularly because NPY-immunoreactive nerve fibres are located in close proximity to immune cells. In addition, there is evidence that NPY may exert pro-inflammatory effects within the intestine [48]. Previous studies have shown that hyperglycaemia is associated with changes in enteric neuronal populations expressing substances involved in inflammatory and neuroimmune processes, including substance P, vasoactive intestinal peptide, and serotonin. Previous studies conducted by the authors demonstrated changes in the proportion of enteric neurons immunoreactive for these substances during streptozotocin-induced diabetes [24,25,26,49,50]. It cannot therefore be excluded that diabetes-associated changes in NPY expression interact with other neuroactive substances and participate in the modulation of local inflammatory responses. Furthermore, recent insights into neuroimmune crosstalk along the gut–brain axis emphasise that neurochemical remodelling within the enteric nervous system should not be considered an isolated local event, but rather as part of complex bidirectional neuroimmune signalling involved in enteric neuroplasticity and inflammatory processes during metabolic disorders [51,52].

It is well established that diabetic enteric neuropathy is closely intertwined with local intestinal inflammation, increased cellular oxidative stress, progressive neuronal dysfunction and loss, and structural tissue remodelling [53]. Although direct quantification of inflammatory cytokines, oxidative stress markers, or apoptotic neuronal death was beyond the primary scope of this morphometric study, the observed increase in the proportion of NPY-immunoreactive neurons should be interpreted within the context of these pathophysiological processes. On the one hand, NPY is known to exert neuroprotective effects that may mitigate oxidative-stress-induced apoptosis, potentially representing an endogenous mechanism counteracting neuronal injury in the diabetic gut [54,55]. On the other hand, sustained alterations in NPY signalling may modulate neuroimmune interactions, contribute to low-grade mucosal inflammation, and induce submucosal microvascular vasoconstriction, thereby potentially participating in structural tissue remodelling and local ischaemic processes [56]. While the lack of complementary molecular and histological markers represents a limitation of the current study, establishing the direct functional relationship between NPY-related neuroplasticity, oxidative injury, and inflammatory cascades remains an important direction for future multi-marker investigations.

The use of prepubertal female pigs in this study was a deliberate design choice aimed at limiting the potentially confounding effects of cyclical fluctuations in female sex hormones on enteric neuroplasticity and thereby increasing experimental homogeneity. Nevertheless, both sex and age may modulate enteric neuronal signalling. Prepubertal animals lack fully mature gonadal steroid activity and may differ from adult animals in their capacity for neuronal plasticity. Data concerning age- and sex-dependent neurochemical remodelling in the porcine enteric nervous system remain scarce; however, available evidence indicates that neuropeptide expression in porcine colonic neurons may vary with age and sex [57]. Therefore, potential age- and sex-dependent differences in NPY-ergic responses should be taken into account when extrapolating the present findings to mature animals, males, or human diabetic populations.

A central question arising from the present findings is whether the observed increase in the proportion of NPY-immunoreactive enteric neurons represents primarily a compensatory and neuroprotective response or a maladaptive alteration potentially contributing to diabetic gastrointestinal dysfunction. On the one hand, at the cellular level, emerging evidence suggests that NPY may act as an endogenous neuroprotective factor in the nervous system and promote neuronal survival [58,59]. Under conditions of metabolic stress, severe hyperglycaemia, and increased oxidative injury characteristic of experimental diabetes, enhanced NPY expression may therefore constitute an adaptive compensatory mechanism aimed at supporting enteric neuronal survival and mitigating neurodegenerative processes [23]. On the other hand, from an organ-level pathophysiological perspective, sustained alterations in NPY-mediated signalling may have potentially detrimental functional consequences [60]. Given that NPY exerts inhibitory effects on gastrointestinal motility and epithelial secretion and has vasoconstrictive effects on submucosal blood vessels, increased NPY signalling could potentially contribute to delayed gastric emptying, intestinal dysmotility, altered mucosal fluid transport, and disturbances in local microvascular perfusion [60,61]. Furthermore, given the ability of NPY to modulate neuroimmune crosstalk and influence the release of inflammatory mediators, sustained changes in NPY signalling may also participate in local intestinal inflammatory processes. Therefore, while increased NPY expression may initially represent an adaptive response aimed at supporting neuronal survival under metabolic stress, its broader functional consequences within the gastrointestinal tract remain uncertain. The observed NPY-related neurochemical plasticity may represent one of the mechanisms contributing to diabetic gastrointestinal dysfunction; however, further functional studies are required to determine whether these changes are predominantly compensatory or contribute to the development and progression of diabetic gastroenteropathy [61,62].

Certain limitations of the present study should, however, be acknowledged. Our investigation primarily focused on quantitative changes within a specific enteric neuronal population, namely NPY-immunoreactive neurons, during relatively short-term hyperglycaemia. Nevertheless, emerging evidence indicates that diabetes-induced alterations in the gut microbiota and dysbiosis may play an important role in intestinal inflammation, neural remodelling, and gastrointestinal complications [52]. Hyperglycaemia-induced oxidative stress may not only affect neuronal neurochemical phenotypes but also increase mucosal permeability, promote dysbiosis, and trigger low-grade intestinal inflammation through complex interactions between the enteric nervous system, immune cells, and the luminal microenvironment. Although direct microbial and immunological profiling was beyond the scope of the present morphometric study, establishing how NPY-related neuroplasticity interacts with alterations in the intestinal microbiota represents an important direction for future translational research.

Furthermore, a methodological limitation of our immunohistochemical approach should be acknowledged. The quantitative analysis was based on the percentage of NPY-immunoreactive neuronal somata relative to the total Hu C/D-immunoreactive neuronal population. Consequently, the observed increase in the proportion of NPY-immunoreactive neurons may reflect increased NPY peptide expression and intracellular accumulation above the threshold of immunohistochemical detection in existing neurons rather than an actual increase in the absolute number of NPY-containing enteric neurons. The present approach therefore does not allow us to distinguish between increased peptide synthesis, altered intracellular accumulation, or enhanced immunohistochemical detectability. Additional molecular analyses of NPY expression would be required to distinguish among these possibilities.

In addition, inflammatory markers, oxidative stress parameters, neuronal loss, and structural alterations of the gastrointestinal wall were not directly evaluated in the present study. Consequently, the relationship between the observed NPY-related neurochemical changes and these processes cannot be established from the present data. Complementary histological and molecular analyses will be required to determine how NPY-related enteric neuroplasticity is associated with inflammation, oxidative stress, neuronal injury, and structural changes during diabetes.

Additionally, individual correlation analyses between blood glucose levels and the proportion of NPY-immunoreactive neurons could not be performed because glycaemic monitoring was recorded as group-averaged weekly measurements. Nevertheless, the diabetic group exhibited a consistent and sustained elevation in blood glucose levels, averaging approximately 17.36–22.26 mmol/L throughout the 6-week experimental period compared with approximately 4.84–5.31 mmol/L in controls. Future studies incorporating individual longitudinal glucose measurements would be valuable to determine whether the observed NPY-related neurochemical changes are quantitatively related to the severity of hyperglycaemia. The exclusive use of prepubertal female pigs should also be considered when interpreting the present findings, as both age and sex may influence enteric neuronal signalling and neurochemical plasticity. Although the use of prepubertal females limited the potentially confounding effects of cyclical fluctuations in female sex hormones and increased experimental homogeneity, the present findings should be extrapolated to mature animals and males with caution. Finally, while the use of five animals per group reflects ethical considerations inherent to large-animal research, the relatively small sample size constitutes an important limitation and may reduce statistical power, particularly after correction for multiple comparisons.

It should be emphasised that throughout the entire experiment, no clinically apparent gastrointestinal disturbances, such as diarrhoea, constipation, or appetite disorders, were observed. Such symptoms occur in many patients with advanced diabetic gastroenteropathy and often develop after prolonged periods of diabetes accompanied by recurrent or sustained episodes of hyperglycaemia. In the present study, the six-week experimental period may have been too short for clinically detectable gastrointestinal manifestations to develop. Nevertheless, the present findings demonstrate that region- and plexus-specific neurochemical changes associated with sustained hyperglycaemia occur at the level of ENS neurons before clinically apparent gastrointestinal dysfunction develops. These early alterations in the neurochemical phenotype of enteric neurons may therefore represent an important component of the initial adaptive and/or pathological response of the ENS to the diabetic environment.

## 4. Materials and Methods

The experiment was conducted on ten sexually immature pigs (Polish Large White breed). The body weight of the animals at the start of the experiment was approximately 20 kg. The animals were kept in the animal quarters of the Faculty of Veterinary Medicine in Olsztyn. The pens in which the animals were kept met the legal standards for this animal species. After a week of acclimatisation, prior to the hyperglycaemic induction procedure, the pigs were randomly and equally divided into two experimental groups (five pigs in a group). The control group (CON) and the group with experimentally induced diabetes (DM).

Diabetes was induced by a single intravenous injection of streptozotocin (STZ), dissolved immediately prior to injection in a citrate buffer solution (pH 4.5), at a dose of 150 mg/kg b.w. (Sigma-Aldrich, St. Louis, MO, USA, S0130), as previously described by Bulc et al. [24]. Due to the rapid breakdown of the beta cells of the pancreatic islets caused by streptozotocin, in order to prevent episodes of sudden and severe hyperglycaemia, the animals in the DM group received prophylactic intravenous injections of 250 mL of a 50% glucose solution per head. In contrast, pigs from the control group received only citrate buffer intravenously. All procedures on animals were conducted in accordance with the Act on the protection of animals for scientific or educational purposes of 15 January 2015 (Journal of Laws of 2015, No. 266), being in force in the Republic of Poland, and were approved by the Local Ethics Committee in Olsztyn (Decision No. 13/2015/DTN, 30 October 2015). Throughout the entire experiment, the pigs from both groups had constant access to water and were fed twice a day (in the morning and evening). The pigs were fed a feed with the following composition (rapeseed meal 6.0%, soybean meal 9.0%, wheat 54.0%, barley 28.5%, other ingredients 2.5%).

Blood for glucose testing was drawn from the marginal vein of the ear by an experienced veterinarian before morning feeding. To this end, the pigs were restrained in accordance with the procedure laid down for this species. Immediately after the blood collection, blood glucose levels were measured calorimetrically using an Accent-200 biochemical analyser (Bimedis, Germany, Hamburg) (wavelengths of 510/670 nm). The measurements were taken in both groups prior to the streptozotocin injection, then again 48 h following the administration of STZ, and then once a week throughout the entire experiment.

On day 43 of the experiment (six weeks following the streptozotocin injection), all the animals were euthanised. The euthanasia procedure was carried out by means of intravenous overdose of pentobarbital (Vetbutal, Biowet, Puławy, Poland). After respiratory arrest, the chest was opened, and a needle was inserted into the left ventricle of the heart to administer 4% buffered paraformaldehyde (pH 7.4). Immediately after perfusion, the following tissues were collected for further testing: the corpus of the stomach, the small intestine (the duodenum: 5 cm behind the pyloric sphincter, the jejunum: 10 cm away from the opening of the duodenal papilla, and the ileum: 5 cm behind the ileocaecal valve), and the descending colon. Fragments of 1 × 1 cm were selected from each collected fragment of the gastrointestinal tract for further testing. The samples were then additionally fixed in the same fixative (10 min), rinsed in phosphate buffer (pH 7.4) for 2 days, with the buffer changed daily, and ultimately placed in an 18% buffered sucrose solution.

In the next step, the collected fragments of individual sections of the gastrointestinal tract were subjected to the procedure of double immunofluorescent staining using the freezing technique (as described previously by Palus and Całka [50]). The prepared blocks were used to create 12 serial sections, each measuring 12 micrometres, which were then placed on microscope slides. The prepared sections were subjected to the following procedure: drying at room temperature for 45 min, followed by rinsing three times (10 min) in 0.1 M phosphate-buffered saline solution (PBS, pH 7.4). The preparations were then blocked in 10% normal goat serum in PBS with 0.3% Triton X-100 (Sigma, St. Louis, MO, USA) and 1% bovine serum albumin (BSA; Sigma, St. Louis, MO, USA) for one hour. They were then rinsed again, three times, in PSB (10 min) and, finally, incubated overnight at room temperature with primary antigenic sera produced against Hu C/D (mouse polyclonal, Invitrogen, Waltham, MA, USA, Cat. No. A-21271, working dilution: 1:1000, used as a pan-neuronal marker) and neuropeptide Y (NPY, rabbit, polyclonal, Abcam, ab43871, working solution: 1:500). After 18 h of incubation at room temperature, the sections were rinsed three times in PBS (10 min) and incubated with a secondary antibody mixture—donkey anti-mouse (Alexa Fluor 488, Thermo Fisher Scientific, Waltham, MA, USA, Cat. No. A21202; working dilution: 1:1000) and donkey anti-rabbit (Alexa Fluor 546, Thermo Fisher Scientific, Waltham, MA, USA, Cat. No. A11074; working dilution: 1:1000)—for one hour at room temperature. Afterwards, the sections were rinsed again in PBS (3 × 10 min) and then sealed with cover slips using a polyethylene glycol/glycerine solution containing DABCO (Sigma, St. Louis, MO, USA). As negative controls, preliminary absorption was performed for neuropeptide antisera with the corresponding antigens, in addition to omission and substitution tests, to eliminate non-specific labelling.

The quantitative analysis was performed using an Olympus BX51 microscope; representative images were taken with a digital monochromatic camera (Olympus XM10, Tokyo, Japan) connected to a PC equipped with the cellSens Dimension Image Processing software (Olympus, Tokyo, Japan). The number of NPY-immunoreactive neurons was calculated as a percentage of all enteric neurons identified by Hu C/D immunoreactivity. For this purpose, at least 500 Hu C/D-immunoreactive neurons with clearly visible nuclei were counted for each animal, separately for each plexus (MP, OSP, and ISP) in each examined segment of the gastrointestinal tract, and considered as 100% of the neuronal population. For each animal, the number of NPY-immunoreactive neurons was expressed as a percentage of the total number of Hu C/D-immunoreactive neurons counted, yielding one percentage value per animal for each plexus and gastrointestinal segment. These animal-level percentage values (*n* = 5 per group) were subsequently used for statistical comparisons between the control and diabetic groups. To avoid double counting the same neuron, sections separated by at least 200 µm were selected for analysis.

The results were statistically analysed using Statistica 13 software (StatSoft Inc., Tulsa, OK, USA) and expressed as mean ± standard deviation (SD). The individual animal was considered the experimental unit, and statistical analyses were performed using one value per animal for each gastrointestinal segment and enteric plexus (*n* = 5 animals per group). Before applying parametric tests, statistical assumptions were formally verified. The distribution of residuals was assessed using the Shapiro–Wilk test, while the homogeneity of variances between groups was evaluated using Levene’s test. In all cases, no significant deviations from these assumptions were observed (*p* > 0.05). Differences between the control and diabetic groups were assessed using the independent Student *t*-test. To account for multiple comparisons across the examined gastrointestinal regions and enteric plexuses, the resulting *p*-values were adjusted using the Holm method. Statistical significance was set at a Holm-adjusted *p* < 0.05, with adjusted significance levels denoted as * *p* < 0.05, ** *p* < 0.01, and *** *p* < 0.001. To provide a clearer biological interpretation of the quantitative differences, fold changes were calculated as the ratio of the mean value in the diabetic group to that in the control group. Effect sizes for pairwise comparisons were evaluated using Cohen’s *d*, with values of approximately 0.2, 0.5, and 0.8 interpreted as small, medium, and large effects, respectively.

## 5. Conclusions

In summary, the present study demonstrates that streptozotocin-induced diabetes, accompanied by sustained hyperglycaemia, is associated with significant changes in the population of NPY-immunoreactive intramural neurons in the porcine gastrointestinal tract. These changes were region- and plexus-specific, with significant increases observed predominantly in the myenteric plexuses of the stomach, jejunum, ileum, and descending colon, while significant changes within the submucosal plexuses were restricted to the descending colon. These findings indicate that NPY-immunoreactive enteric neurons participate in the neurochemical plasticity of the enteric nervous system associated with diabetic conditions. The observed changes may represent an adaptive response to metabolic stress and/or contribute to altered neural regulation of gastrointestinal function during diabetes; however, their precise functional significance remains to be established. Further studies are required to determine the functional consequences of altered NPY-related signalling and its potential relevance to the development of diabetic gastroenteropathy.

## Figures and Tables

**Table 1:** Blood glucose concentrations in control and streptozotocin-treated pigs during the experimental period (mean ± SEM).

Date	Control Group (mmol/L)	SEM ±	Experimental Group (mmol/L)	SEM ±
Before streptozotocin injection	5.01	0.10	5.03	0.10
1 week after streptozotocin injection	5.08	0.10	17.36	0.38
2 weeks after streptozotocin injection	4.91	0.18	20.72	0.24
3 weeks after streptozotocin injection	5.19	0.06	21.58	0.27
4 weeks after streptozotocin injection	5.31	0.12	20.08	0.09
5 weeks after streptozotocin injection	4.84	0.32	22.26	1.21
6 weeks after streptozotocin injection	5.20	0.10	21.45	1.11

## Data Availability

The original contributions presented in this study are included in the article. Further inquiries can be directed to the corresponding author.
